# Troxerutin Delays Skin Keratinocyte Senescence Induced by Ionizing Radiation Both In Vitro and In Vivo

**DOI:** 10.1111/jocd.16584

**Published:** 2024-09-18

**Authors:** Juping Chen, Jinghui Yang, Jiang Ma, Xiaoming Sun, Yuxuan Wang, Changjiao Luan, Jiaxiao Chen, Weili Liu, Qing Shan, Xingjie Ma

**Affiliations:** ^1^ Department of the Central Laboratory, Department of Intensive Care The Affiliated Hospital of Yangzhou University, Yangzhou University Yangzhou China; ^2^ Department of Dermatology The Affiliated Hospital of Yangzhou University, Yangzhou University Yangzhou China; ^3^ Department of Lung The Third People's Hospital of Yangzhou Yangzhou China; ^4^ Department of Geriatrics The Affiliated Hospital of Yangzhou University, Yangzhou University Yangzhou China

**Keywords:** age, aging skin, cell signaling, DNA damage, keratinocyte, mitochondria dysfunction, skin cellular senescence, Trx

## Abstract

**Backgrounds:**

With the increasing demand for beauty and a healthy lifespan, studies regarding anti‐skin aging have drawn much more attention than ever before. Skin cellular senescence, the primary cause of skin aging, is characterized by a cell cycle arrest in proliferating cells along with a senescence‐associated secretory phenotype (SASP), which can be triggered by various internal or external stimuli.

**Aims:**

Recent studies have made significant progress in the fields of anti‐senescence and anti‐aging. However, little is known about the roles and functions of natural compounds, particularly flavonoids, in skin cellular senescence studies.

**Methods:**

In this study, using strategies including ionizing radiation (IR), senescence‐associated β galactosidase assay (SA‐β‐Gal), immunofluorescence (IF), flow cytometry, PCR array, as well as in vivo experiments, we investigated the effects and roles of troxerutin (Trx), a natural flavonoid, in skin keratinocyte senescence.

**Results:**

We found that Trx delays skin keratinocyte senescence induced by IR. Mechanistically, Trx protects the skin keratinocyte cells from senescence by alleviating reactive oxygen species (ROS) accumulation, mitochondrial dysfunction, and DNA damage caused by IR. In addition, Trx was also proved to relieve skin senescence and SASP secretion in vivo induced by IR stimulation.

**Conclusions:**

Altogether, our findings pointed to a new function of Trx in delaying stress‐induced skin keratinocyte senescence, and should thus provide theoretical foundations for exploring novel strategies against skin aging.

## 1 Introduction

Cellular senescence is caused by diverse stimulations including telomerase shortening, oxidative stresses, DNA damages, and oncogenic activation, contributing to multiple pathophysiological outcomes, such as embryonic development, wound healing, inflammation, aging, and age‐associated disorders [1]. Comparing to normal proliferating cells, senescent cells can be recognized by their characteristics including senescence‐associated secretory phenotype (SASP), anti‐apoptotic abilities, increased cyclin‐dependent kinase inhibitors, senescence‐associated β galactosidase (SA‐β‐Gal) activity, etc. [2]. Ionizing radiation (IR) induces the overproduction of reactive oxygen species (ROS), leading to persistent oxidative stress, mitochondrial dysfunction, and ultimately cellular senescence or apoptosis [3]. With the increasing importance of radiotherapy in oncological treatment, the frequency of people's exposure to IR is increasing. Skin tissues, as a protective barrier against external environment, are particularly vulnerable to IR‐induced toxicities, including skin cellular senescence and aging [4].

The global population of people over 60 years old is rapidly increasing and is projected to reach approximately 1/6 of the total global population by 2030 [5]. Consequently, studies on anti‐aging and anti‐age‐related diseases have emerged as one of the biggest issues and also one of the most attractive fields worldwide. Currently, there are three main aspects regarding anti‐aging studies: (1) Identifying genes, pills, or natural compounds to delay senescence of targeted cells, for example, knockdown of p53 or inhibit its downstream p21 contributes to bypass of fibroblast cellular senescence [6, 7]. (2) Screening for therapeutic tools to selectively kill senescent cells, such as Dasatinib and Quercetin (D + Q), ABT‐263/navitoclax, and senescence‐specific killing compound 1 (SSK1) [8]. (3) Applying clinical drugs or specific inhibitors to eliminate or suppress SASP production without affecting the cells, exampled by rapamycin and ruxolitinib [9]. Nevertheless, there are no sufficient and effective strategies in anti‐skin aging studies till now.

Troxerutin (Trx), a natural flavonoid derived from hydroxyethylation of rutin extracted from *Sophora japonica* [10], is widely known for its antioxidant and anti‐inflammatory effects in various cellular and murine models [11]. For example, Trx has been demonstrated to alleviate therapy‐induced cardiotoxicity [12] and neurodegenerative diseases [13]. In addition, Trx was also proved to ameliorate methotrexate (MTX)‐induced nephrotoxicity renal injury in male Wistar albino rats through inhibiting NF‐κB‐mediated inflammation, repressing apoptosis as well as activating autophagy [14]. However, little is known about the effects of Trx on skin cellular senescence.

Here, in this study, we utilized Trx to treat IR‐induced senescent human keratinocyte HaCaT cells and IR‐exposed mice dorsal skin tissues, thus aiming to investigate the effects and regulatory mechanisms of Trx on skin keratinocyte senescence both in vitro and in vivo.

## 2 Results

### 2.1 Trx Delays Skin Keratinocyte Senescence Induced by IR

To determine the optimal dose of IR for inhibiting HaCaT proliferation, three doses of IR (4Gy, 6Gy, and 8Gy) were used to expose cells. Results showed that 6Gy was sufficient to induce cell proliferation arrest (Figure S1a). Subsequently, three concentrations of Trx (1, 5, and 10 μM) were tested before exposing to 6Gy IR. Results indicated that a concentration of 5 μM Trx was adequate to reverse the inhibited proliferation of HaCaT cells caused by 6Gy IR (Figure S1b).

**FIGURE 1:**
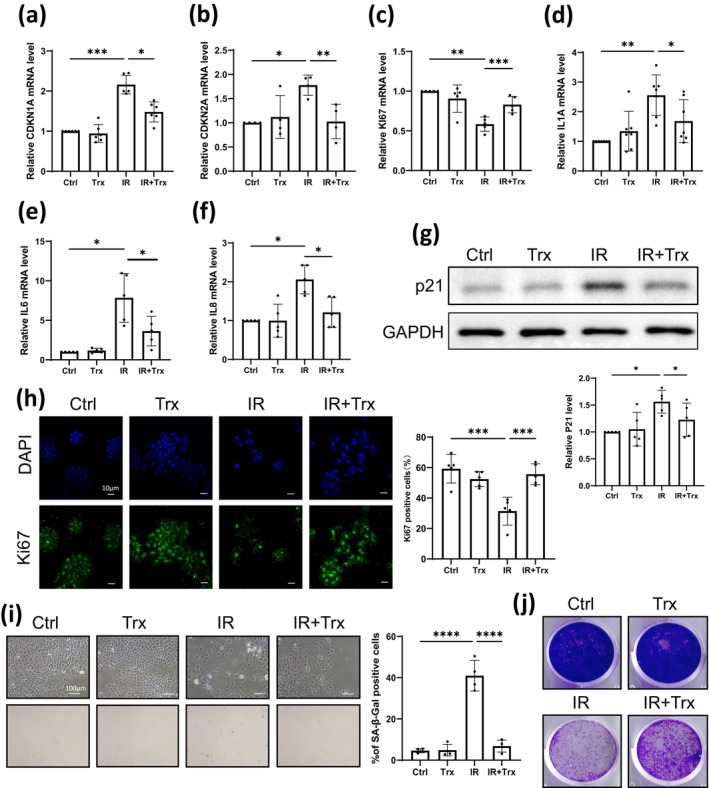
Trx delays skin keratinocyte senescence induced by IR. HaCaT cells were pretreated with Trx 24 h before exposing to 6Gy IR. (a–f) Three days after IR exposure, cells were collected and mRNA levels of indicated genes were detected using RT‐qPCR (mean ± SD, *n* = 6 for (a), *n* = 4 for (b), *n* = 5 for (c, e, f), and *n* = 7 for (d)). (g) Four days after IR exposure, the protein levels of p21 and GAPDH were detected by western blot; representative images and quantifications were shown (mean ± SD, *n* = 5). (h) Four days post IR exposure, IF staining against Ki67 was performed. Left panels showed representative images and right panels showed the percentage of Ki67 positive cells (representative of three independent repeats with mean ± SD, *n* = 5). (i) Four days post IR exposure, cells were stained with SA‐β‐Gal solution; representative photos were shown in the right panel and the percentage of SA‐β‐Gal positive cells was indicated in the left panel (representative of three independent repeats with mean ± SD, *n* = 4). (j) Five days after IR exposure, cells were stained with crystal violet (representative of three independent repeats). Statistical analysis: Paired one‐way ANOVA test for (a–g) and unpaired one‐way ANOVA test for (h, i) (*p < 0.05, **p < 0.01, ***p < 0.001, and ****p < 0.0001).

Trx (5 μM) was then administered to HaCaT cells 24 h prior to IR exposure (6Gy doses), which was applied to establish the HaCaT cellular senescence model, to evaluate the effects on senescence. The results indicated that the induction of senescence markers *CDKN1A*, *CDKN2A*; proliferating marker Ki67 (both mRNA and protein levels); and SASP factors *IL1A*, *IL6*, and *IL8* by IR were all significantly reversed after adding Trx (Figure 1a–f,h). In addition, the upregulated expression of p21, which was encoded by CDKN2A, by IR was also alleviated upon Trx treatment (Figure 1g). SA‐β‐Gal staining revealed that Trx was able to repress the increased percentage of senescent cells induced by IR (Figure 1i). Crystal violet staining indicated that Trx could inhibit the proliferation arrest caused by IR (Figure 1j). Collectively, these data illustrated that Trx delayed skin keratinocyte senescence phenotypes induced by IR.

### 2.2 Trx Rejuvenates MMP Drop and ROS Accumulation Induced by IR

**FIGURE 2:**
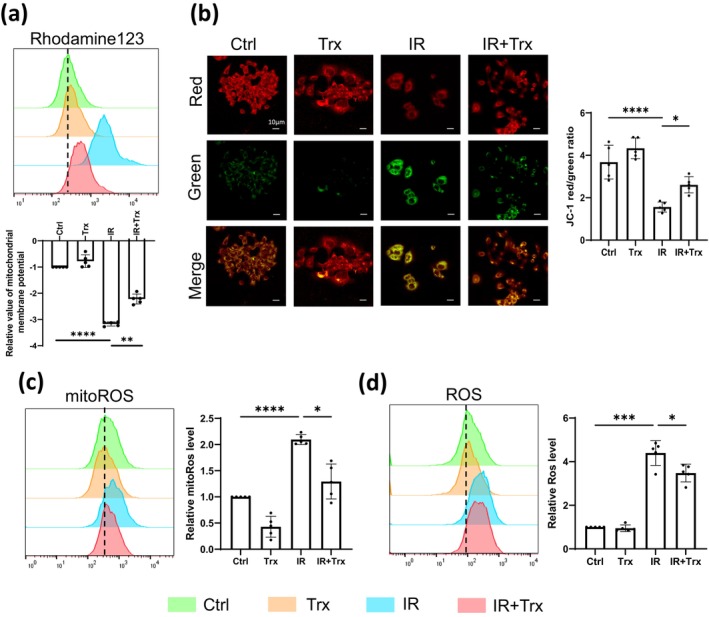
Trx rejuvenates MMP drop and ROS accumulation induced by IR. HaCaT cells were pretreated with Trx 24 h before exposing to 6Gy IR. Four days after IR exposure, cells were analyzed with flow cytometry using MMP indicator Rhodamine123 (a), mitoSOX RED mitochondrial (mitoROS) indicator (c), or dihydroethidium (ROS) reporter (d). Representative images and the biological repeats with mean ± SD were shown (*n* = 5). (b) Four days after IR exposure, cells were incubated with JC‐1 probe, and the JC‐1 red/green ratio was calculated (representative of three independent repeats with mean ± SD, *n* = 5). Statistical analysis: Paired one‐way ANOVA test for (a, c, d) and unpaired one‐way ANOVA test for (b) (*p < 0.05, **p < 0.01, ***p < 0.001, and ****p < 0.0001).

To investigate the mechanisms through which Trx inhibits stress‐induced skin keratinocyte senescence, we interrogated literatures and found Trx is involved in the regulation of mitochondrial oxidative stresses [12]. We therefore examined the mitochondrial membrane potential (MMP) and interestingly found out that IR induced a drop of MMP in HaCaT cells, whereas treatment of these cells with Trx was sufficient to rejuvenate the decreased MMP as evidenced by Rhodamine123 (Figure 2a) and JC‐1 probe (Figure 2b). As MMP drop always triggers the increasing of mitochondrial ROS (mitoROS) level [15], we subsequently detected and observed that the accumulation of both mitoROS and ROS production promoted by IR were abolished after treating the cells with Trx (Figure 2c,d). In summary, these data illustrated that Trx restores MMP drop and inhibits ROS accumulation induced by IR.

### 2.3 Trx Alleviates Mitochondrial Dysfunction During Skin Keratinocyte Senescence

**FIGURE 3:**
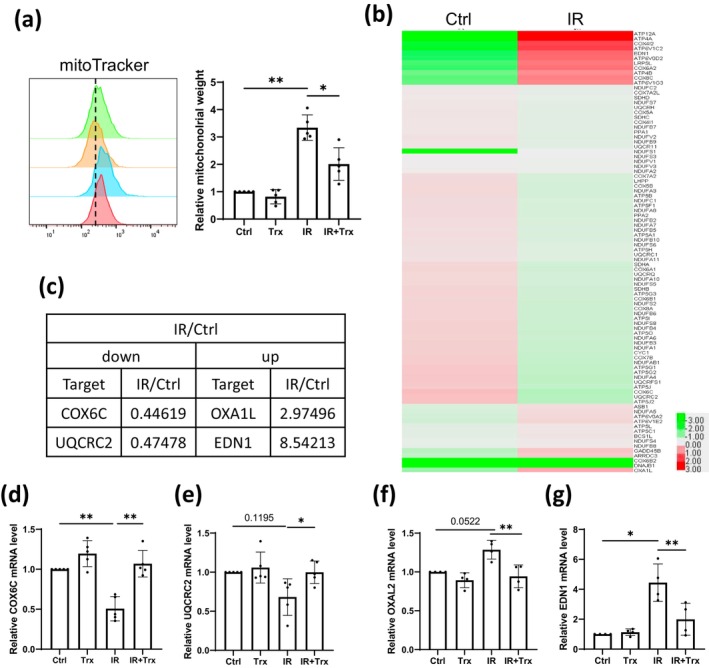
Trx alleviates mitochondrial dysfunction during skin keratinocyte senescence. (a) HaCaT cells were pretreated with Trx 24 h before exposing to 6Gy IR. Four days after IR exposure, cells were analyzed with flow cytometry using mitoTracker deep red FM. Representative images and the biological repeats with mean ± SD were shown (*n* = 5). (b) HaCaT cells were exposed to 6Gy IR. Four days later, cells were analyzed with PCR assay against mitochondrial energy metabolism, and different expressed genes were shown. (c) Top two down‐ and upregulated genes from PCR array were shown. (d–g) HaCaT cells were pretreated with Trx 24 h before exposing to 6Gy IR. Three days after IR exposure, cells were collected and mRNA levels of indicated genes were detected using RT‐qPCR (mean ± SD, *n* = 5 for (d, e) and *n* = 4 for (f, g)). Statistical analysis: Paired one‐way ANOVA test (*p < 0.05 and **p < 0.01).

Furthermore, as overproduced ROS in the cells may lead to mitochondrial dysfunction [16], we firstly assessed the mitochondrial mass using mitoTracker. The results showed that IR facilitated an elevation of mitochondrial mass, while this increase can be diminished by Trx treatment (Figure 3a), indicating alternations in morphology caused by IR in mitochondria. We hence performed a PCR array against mitochondrial energy metabolism in HaCaT cells treated with or without IR. A total of 90 energy metabolism‐associated genes were tested, and as expected, a majority of them (64 in 90) were downregulated upon IR exposure (Figure 3b). We then selected the top two downregulated genes (cytochrome c oxidase subunit 6c, (COX6C) and ubiquinol‐cytochrome c reductase core protein 2 (UQCRC2)) and the top two upregulated genes (OXA1L mitochondrial inner membrane protein (OXA1L) and endothelin 1 (EDN1)) from the array for further verification (Figure 3c). Interestingly, Trx reverted the expression of IR‐repressed *COX6C* and *UQCRC2* as well as IR‐induced *OXA1L* and *EDN1* (Figure 3d–g). Altogether, these findings demonstrated that Trx alleviated IR‐induced mitochondrial dysfunction during skin keratinocyte senescence.

### 2.4 Trx Alleviates the DNA Damage Induced by IR

**FIGURE 4:**
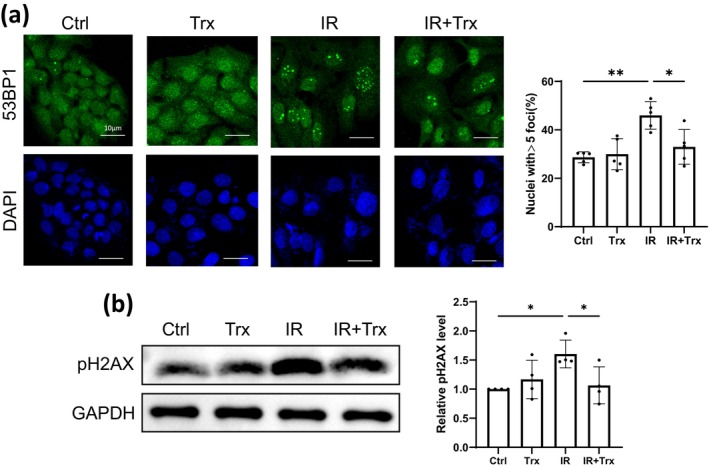
Trx alleviates the DNA damage induced by IR. HaCaT cells were pretreated with Trx 24 h before exposing to 6Gy IR. (a) Four days post IR exposure, IF staining against 53BP1 was conducted. The left panels showed representative images and the right panels showed the percent of nuclei with ≥ 5 foci (representative of three independent repeats with mean ± SD, *n* = 5). Four days after IR exposure, the protein levels of pH2AX and GAPDH (b) were detected by western blot; representative images and quantifications were shown (mean ± SD, *n* = 4). Statistical analysis: Unpaired one‐way ANOVA test for (a) and paired one‐way ANOVA test for (b) (*p < 0.05 and **p < 0.01).

Mitochondrial dysfunction is known to promote DNA damage [17]. Therefore, we checked for DNA damage using two markers: 53BP1 and p‐H2AX. As shown in Figure 4a,b, Trx was able to decrease the DNA damage promoted by IR. We thereby summarized that Trx remitted the DNA damage induced by IR.

### 2.5 Trx Delays Skin Senescence Phenotype Induced by IR In Vivo

**FIGURE 5:**
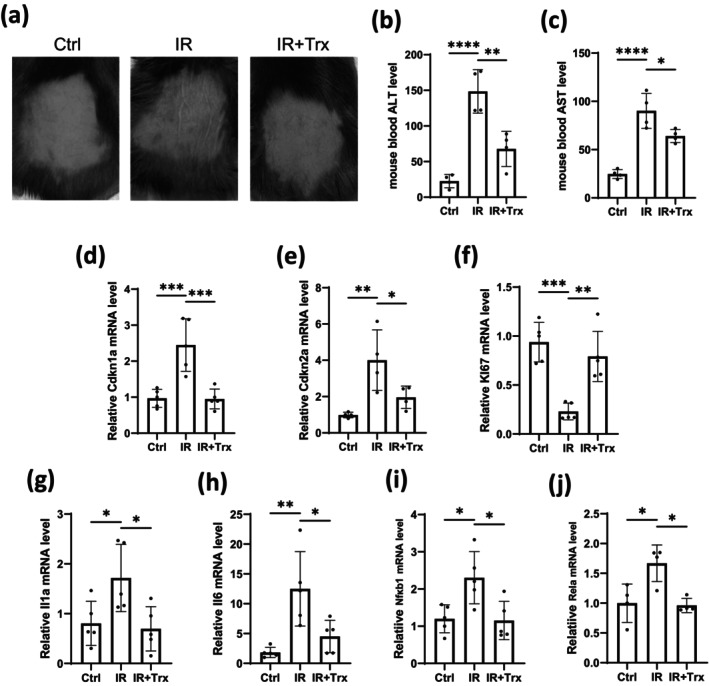
Trx delays skin senescence phenotype induced by IR. Mice were treated with Trx prior to being exposed to twice doses of 5Gy IR on Day 1 and Day 4. Ten days later, (a) the skin phenotype was shown. The AST (b) and ALT (c) levels in the blood were checked (mean ± SD, *n* = 4). (d–j) Dorsal skin tissues were collected and mRNA levels of indicated genes were detected using RT‐qPCR (mean ± SD, *n* = 5 for (e, g–j) and *n* = 4 for (f, k)). Statistical analysis: Unpaired one‐way ANOVA test (*p < 0.05, **p < 0.01, ***p < 0.001, and ****p < 0.0001).

As Trx has been proved to delay skin keratinocyte senescence induced by IR, we next wondered whether or not Trx plays roles in skin senescence in vivo. We established an IR‐triggered dorsal skin senescence model as previously described [18]. Upon IR exposure, the skin exhibited wrinkling, desiccation, and hypoelasticity, whereas Trx inhibited these phenotypes (Figure 5a). Additionally, levels of aspartate transaminase (AST) and alanine aminotransferase (ALT) in the blood were measured, with results showing that Trx repressed the IR‐facilitated elevation of AST and ALT, indicating potential protection against IR‐induced liver damage (Figure 5b,c). Notably, the upregulation of senescence markers *Cdkn1a* and *Cdkn2a* induced by IR‐induced was partially reversed upon Trx treatment (Figure 5d,e). Similarly, downregulation of proliferating marker *Ki67* and induction of SASP factors *Il1a*, *Il*6, *Nf‐κb1*, and *Rela* were also partially reverted by Trx treatment (Figure 5f–j). In addition, the dorsal skin thickness was measured after H&E staining, and IR was found to promote thinning of the skin, which can be reversed by Trx (Figure S2). Taken together, Trx was shown to delay skin senescence phenotype and SASP secretion induced by IR in vivo.

## 3 Discussion

In this study, it was demonstrated that Trx delays skin keratinocyte senescence by alleviating intracellular ROS accumulation, mitochondrial dysfunction, and DNA damage caused by IR exposure. Furthermore, IR stimulation promoted skin senescence phenotype and SASP secretion in vivo can also be abolished upon Trx treatment.

The skin acts as a barrier organ against external stimulations of the body. Along with economy development and increased personal awareness, more attentions have been paid to the health and anti‐aging of the skin. Skin aging, primarily driven by cellular senescence, is typically displayed as a reduced barrier integrity, sagging, wrinkling, thinning of the epidermis, and age spots [19]. A number of studies have focused on investigating skin cellular senescence and anti‐skin aging strategies. For instances, deficiency of growth arrest and DNA damage inducible beta (Gadd45b) was proved to promote senescence and aging phenotypes in mouse skin [20]. Doxercalciferol, an analog of vitamin D2, inhibited the ultraviolet radiation B (UVB)‐induced NF‐κB and mitogen‐activated protein kinase (MAPK) senescence pathways in HaCaT cells as well as skin photoaging and damage in mice [21]. Additionally, hesperetin, a CDGSH iron–sulfur domain‐containing protein 2 (CISD2) activator, was demonstrated to enhance mitochondrial function, alleviate UVB‐induced injury in human keratinocytes, as well as delay skin aging and rejuvenate naturally aged skin in vivo [22].

Here we found that Trx alleviated the IR‐triggered mitoROS, intracellular total ROS accumulation, and DNA damage. Although Trx was originally known for its antioxidant and anti‐inflammatory functions in different pathophysiology of diseases, it also showed protective effects in gentamycin‐induced acute kidney injury through decreasing urinary albumin level, blood urea nitrogen, and serum creatinine as well as improving glomerular filtration rate [23]. In addition, in ischemic brain tissues, combination treatment of Trx with cerebroprotein hydrosylate (TCHI) contributed to decreasing lactic acid, while increasing superoxide dismutase (SOD) activity and lactate dehydrogenase (LDH) level [24]. Moreover, simultaneous treatment of human gastric cancer cells with Trx and 5‐fluorouracil (5‐FU) increased the sensitivity of cancer cells to 5‐FU, thus inhibiting the cell proliferation rate and promoting apoptosis [25]. Intriguingly, Trx was also found to repress the IR‐induced apoptosis in thymocytes by inhibiting PTEN and activating Akt signaling [26]. Here we are the first to report that Trx was able to inhibit the overproduction of ROS and DNA damage in skin keratinocytes cells induced by IR. Due to its abundant beneficial biological functions, Trx may be a promising natural product for treating various disorders.

Our data also demonstrated that Trx played a role in mitochondrial dysfunction and SASP secretion during skin keratinocyte senescence. Mitochondrial dysfunction is mainly described as a drop of MMP, decreasing of ATP production and respiration rate [27]. In addition to the PCR array analysis and confirmation of genes expressions in this study, further efforts are needed to elucidate the precise roles and regulatory pathways of Trx in mitochondrial dysfunction. Interestingly, we found that Trx inhibited SASP secretion triggered by IR. Therefore, treatment with Trx or related agents may be useful strategies against skin inflammation and age‐associated skin pathologies. Additionally, while previous reports have shown that Trx alleviated ulcerative colitis [28], lipopolysaccharide‐induced sepsis [29], and cognitive deficits [30], our in vivo experiment indicated that Trx protected against IR‐induced skin senescence. However, although Trx showed a protective effect toward senescent keratinocyte cells and skin tissues, it remains to be investigated whether or not Trx could control senescence of another two most studied skin cell types: melanocytes and fibroblasts, as well as the importance of keratinocyte senescence during skin aging.

In conclusion, our study demonstrated that Trx protects against IR‐promoted ROS accumulation, mitochondrial dysfunction, DNA damages, as well as ultimately skin senescence and SASP secretion. This work revealed that the natural anti‐senescence product Trx has a great potential for application in anti‐stress induced skin aging. However, whether or not Trx also plays key roles in naturally aged skin and age‐related skin disorders remains to be investigated.

## 4 Materials and Methods

### 4.1 Cell Culture and Treatments

The human skin keratinocyte cell line HaCaT (Stem Cell Bank of Chinese Academy of Sciences) was grown in RPMI 1640 basic medium with L‐Glutamine supplemented with 10% fetal bovine serum (FBS, Procell) and 1% penicillin/streptomycin (Solarbio). Cells were maintained at 37°C under 5% CO_2_. Cells were regularly tested for mycoplasma contamination and experiments were conducted until cells were confirmed to be mycoplasma‐free. Trx (MCE) was used at 5 μM.

### 4.2 RNA Extraction, Reverse Transcription, and Quantitative PCR

Total RNA was extracted using TRNzol (TIANGEN) according to the manufacturer's instructions. Complementary DNAs were synthesized using PrimeScript RT Master Mix (TAKARA) and diluted 10 times for quantitative PCR (qPCR) analysis. A total of 10 μL qPCR mix contained 5 μL of 2×Hieff qPCR SYBR Green Master Mix (No Rox) (YEASEN), 0.4 μL of forward and reverse primers (10 μM), 1.5 μL cDNA, and ddH_2_O up to 10 μL. The reaction was run in a cFX96 Touch Thermocycler (Bio‐Rad) machine with following protocol: 95°C 5 min, following with 40 cycles of 95°C 10 s and 60°C 30 s, and a melt curve from 65°C to 95°C with an increment 0.5°C for 5 s. Sequences of primers were listed in Tables S1 and S2. Duplicate was performed for each sample and the comparative CT (ΔΔCT) method was used for the calculation. mRNA levels were calculated after normalizing to the mean of PGK1 and HPRT1 housekeeping genes (for human samples) or the GAPDH (for mice samples).

### 4.3 Animals

C57BL/6 mice (6–8 weeks) used in this study were provided by the Institute of Comparative Medicine of Yangzhou University. Mice were maintained in a pathogen‐free environment, subjected to a 12‐h light and 12‐h dark cycle, within temperature‐controlled conditions at a humidity level of 45.5%, with unrestricted access to food and water. Mice groups, treatment order, and locations were all randomized. The study was based on the Principles of Laboratory Animal Care (NIH publication No. 85Y50, revised 1996) and was approved by the Laboratory Animal Ethics Committee of Yangzhou University. In brief, sodium pentobarbital (50 mg/kg) was used to anesthetize the mice, and the dislocating cervical vertebrae method was applied to sacrifice the mice under deep anesthesia. The dorsal skin samples were then separated and stored at −80°C for further analysis.

### 4.4 Ionizing Radiation Exposure

For in vivo experiments, 20 mg/kg Trx (1 mg/mL) was administrated every 2 days by intraperitoneal injection starting 4 days before IR exposure. The dorsal skin of the mice was exposed to IR using the linear accelerator Artiste (VARIAN, USA) with 5Gy twice on Day 1 and Day 4 (6‐MV X photons, 2Gy/min). 10 days after the first exposure, mice were sacrificed and dorsal skin specimens were isolated for further experiments. The control group received exactly the same treatment except for being exposed to 0Gy IR.

For in vitro experiments, cells in 12‐well plates or 6‐well plates were exposed to 6Gy IR, then the medium was immediately changed after exposure. The control group underwent identical conditions except for receiving 0Gy IR. Cells were then maintained in an incubator for further experiments.

### 4.5 Immunofluorescence

The 12‐well plates with coverslips (Solarbio) were used to seed HaCaT cells. Cells on the coverslips were washed with 1×PBS and were fixed with cold methanol for 10 min at −20°C. Subsequently, the cells were blocked in PBS‐Tween containing 20% FBS for 30 min and were incubated with primary antibodies overnight at 4°C (Table S3). On the following day, the cells were washed and incubated with secondary antibodies for 1 h at room temperature. Nuclei were stained with DAPI Fluoromount G (SouthernBiotech, USA). Images were acquired with the Nikon fluorescence microscope and NIS software (Nikon, Japan), and the indicated fluorescence intensity was obtained using Image J software.

### 4.6 Senescence‐Associated β Galactosidase Assay and Crystal Violet Staining

For SA‐β‐Gal staining: HaCaT cells in 12‐well plates were washed once with 1×PBS and then were fixed in 2% formaldehyde in 0.2% glutaraldehyde for 5 min. SA‐β‐Gal staining solution was used and cells were incubated overnight at 37°C in the dark under CO_2_‐free condition [31]. The following day, the SA‐β‐Gal staining solution was removed from the cells and the plates were rinsed with and maintained in 1×PBS for taking photos.

For crystal violet staining: cells in 12‐well plates were washed with PBS, then fixed with 3.7% formaldehyde for 15 min, and stained overnight with 0.05% crystal violet staining solution. The following day, the staining solution was removed, and cells were rinsed and scanned after drying.

### 4.7 Western Blot Analysis

HaCaT cells were lysed in RIPA buffer and the protein concentration was determined using BCA kit (Thermo Fisher Scientific). Equal amounts of proteins from each sample were separated with SDS‐PAGE electrophoresis after boiling. Samples were then blotted onto a nitrocellulose membrane (Bio‐Rad). Membranes were blocked with TBS‐tween blocking buffer (5% milk powder and 0.05% Tween‐20 in PBS), and then incubated with primary antibodies (Table S3) at 4°C overnight. The following day, after washing, the membranes were incubated with peroxidase‐conjugated secondary antibodies for 1 h at room temperature. The membranes were then detected using ECL Plus chemiluminescent system (Bio‐Rad) according to manufacturer's instructions.

### 4.8 Flow Cytometry

HaCaT cells with indicated treatment were incubated with probes (Table S3) at 37°C for half an hour. After that, the cells were washed and maintained in PBS for analysis in flow cytometry. Data was acquired with CytExpert for DxFLEX (BECKMAN COULTER, USA) and was analyzed with FlowJo v10 software.

### 4.9 PCR Array

Total RNA was isolated from HaCaT cells and cDNAs were synthesized. The gene expression profiling of mitochondrial energy metabolism was carried out with cDNAs using Gene Expression PCR Array kit (WcGene Biotech) according to the manufacturer's protocols.

### 4.10 AST and ALT Measurement

Venous blood samples were collected and the supernatant was isolated after 3000 rpm centrifugation for 15 min at room temperature. The AST and ALT kits (Jiancheng Biotech) were applied to measure the AST and ALT levels in serum according to the manufacturer's instructions.

### 4.11 Statistical Analysis

GraphPad Prism v.9.0 was used for statistical analysis. Generally, D'Agostino and Pearson's normality test was used to evaluate the data before any analysis. One‐way or two‐way ANOVA and paired or unpaired (depend on the conditions) Tukey's multiple comparison tests were applied for data that were normal distributed. Kruskal–Wallis and Dunn's multiple comparison tests were performed to determine statistical significance for data with non‐normal distribution. Results were shown with mean ± SD (*n* was indicated in figure legends; ns, non‐significant; *p < 0.05; **p < 0.01; ***p < 0.001; and ****p < 0.0001).

## Author Contributions

X.M., Q.S., and W.L. supervised this project and designed all the experiments. J.C., J.Y., J.M., X.S., Y.W., C.L., and J.C. performed experiments. Ju.C., J.Y., and X.M. analyzed the data. X.M. wrote the manuscript with input from all authors.

## Ethics Statement

All in vivo studies were approved by the Science and Technology Commission of the Affiliated Hospital of Yangzhou University (2020‐YKL04‐Y003).

## Conflicts of Interest

The authors declare no conflicts of interest.

## Supporting information


Data S1.

## Data Availability

The data that support the findings of this study are available from the corresponding author upon reasonable request.
