# Supplementary material for: Troxerutin Delays Skin Keratinocyte Senescence Induced by Ionizing Radiation Both In Vitro and In Vivo
Source: J Cosmet Dermatol. 2024 Sep 18;24(1):e16584. doi: 10.1111/jocd.16584 (PMC11743059; doi:10.1111/jocd.16584)
Supplement: Supplementary file 1 — Data S1. [file JOCD-24-e16584-s001.pdf]

Figure S1. Chen et al.

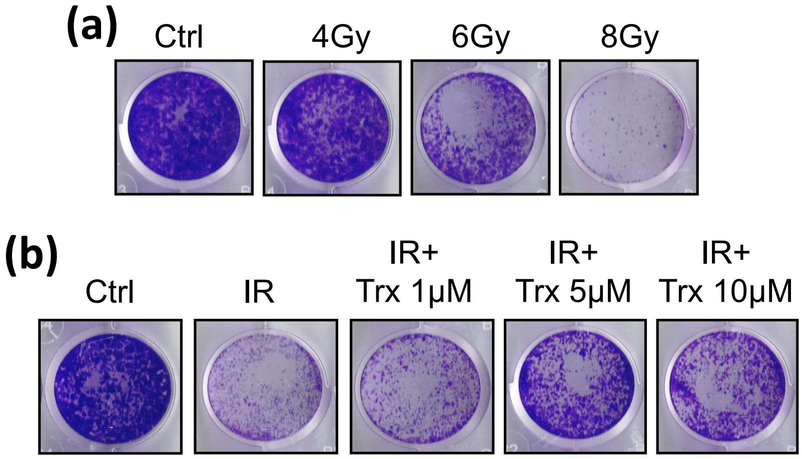

Figure S1 Crystal violet staining of HaCaT cells after IR exposure. (a) HaCaT cells were exposed to different doses of IR. Five days later, cells were stained with crystal violet (representative of three independent repeats). (b) HaCaT cells were treated with different concentrations of Trx 24h before IR exposure and then subjected to 6 gy IR. Five days after IR exposure, cells were stained with crystal violet (representative of three independent repeats).

Figure S2. Chen et al.

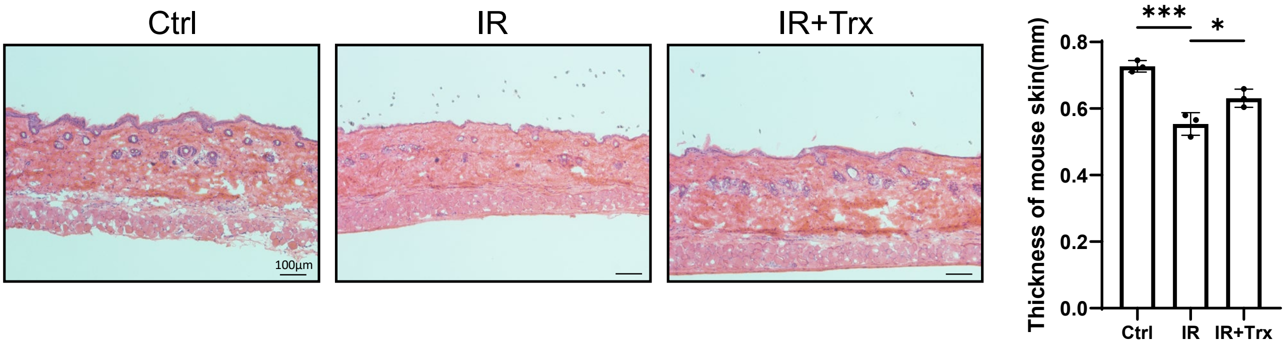

Figure S2 H&E staining of the exposed skin was shown and thickness of skin was measured (mean ± SD, n=3). Statistical analysis: unpaired one-way ANOVA test (*P < 0.05 and ***P < 0.001).

Table S1 Human primers used for RT-qPCR in this study.

Gene symbol	Forward primers	Reverse primers
PGK1	GCCAAGTCGGTAGTCCTTATG	CCCAGCAGAGATTTGAGTTCTA
HPRT1	CGAGATGTGATGAAGGAGATGG	TTGATGTAATCCAGCAGGTCAG
CDKN1A	CGGAACAAGGAGTCAGACATT	AGTGCCAGGAAAGACAACTAC
CDKN2A	GCACATTCATGTGGGCATTT	GACTCAAGAGAAGCCAGTAACC
Ki67	GACCTCAAACTGGCTCCTAATC	GCTGCCAGATAGAGTCAGAAAG
IL1A	TGTGACTGCCCAAGATGAAG	CGTGAGTTTCCCAGAAGAAGAG
IL6	GGAGACTTGCCTGGTGAAA	CTGGCTTGTTCCTCACTACTC
IL8	CTTGGCAGCCTTCCTGATTT	GGGTGGAAAGGTTTGGAGTATG
COX6C	GTTTCGTGTGGCTGATCAAAG	CTCTGAAAGATACCAGCCTTCC
UQCRC2	CTCAGCAGCCATTTGATGTTTC	TGTGGCCTGGGAGATAGTATAA
OXA1L	CTGACCTTCAGTGGATGAGAAA	GGAGGAGAGCCAGTACATAAAC
EDN1	GCAGAAACACACAGTCACATTC	CCTTAGGACCTTCGTCAGAAAC

Table S2 Murine primers used for RT-qPCR in this study.

Gene symbol	Forward primers	Reverse primers
Gapdh	AACAGCAACTCCCACTCTTC	CCTGTTGCTGTAGCCGTATT
Cdkn1a	GCTGGGTGGTCTTTGTGTA	TTAGCTCTGCTCTTGGGATTG
Cdkn2a	GTTCCTTGCCACTTCTTACCT	TCATCCTAGCTGGCCTTAGA
Ki67	CCACACAGATGCCCTGTAAT	CTCTACTTTCCCACGTCTTGTC
Il1a	GCTTGAGTCGGCAAAGAAATC	GAGAGATGGTCAATGGCAGAA
Il6	GTCTGTAGCTCATTCTGCTCTG	GAAGGCAACTGGATGGAAGT
Nfkb1	AGACATCCTTCCGCAAACTC	TAGGTCCTTCCTGCCCATAA
Rela	CCGACTTGTTTGGGTGATCT	TCCGTCTCCAGGAGGTTAAT

Table S3 Antibodies and probes used in this study.

Application	Name	Reference	Company	Dilution
IF	53BP1	4937	Cell Signaling Technology	1:200
IF	Ki67	sc-23900	santa cruz	1:200
WB	p21	sc-6246	santa cruz	1:300
WB	pH2AX	sc-517348	santa cruz	1:300
WB	GAPDH	sc-365062	santa cruz	1:500
FACS	Rhodamine123 (2mM)	R8004	Sigma	1:3000
FACS	mitoSOX RED mitochondrial (1µg/µl)	M36008	Invitrogen	1:166.67
FACS	Dihydroethidium (5µM)	D7008	Sigma	1:100
FACS	mitotracker deep red FM	M22426	ThermoFisher	1:1000
